# Ventricular arrhythmia events in heart failure patients with cardiac resynchronization therapy with or without a defibrillator for primary prevention

**DOI:** 10.1002/joa3.12795

**Published:** 2022-10-31

**Authors:** Toshihiro Nakamura, Koji Fukuzawa, Kunihiko Kiuchi, Mitsuru Takami, Yusuke Sonoda, Hiroyuki Takahara, Kazutaka Nakasone, Kyoko Yamamoto, Yuya Suzuki, Ken‐ichi Tani, Hidehiro Iwai, Yusuke Nakanishi, Mitsuhiko Shoda, Atsushi Murakami, Shogo Yonehara, Ken‐ichi Hirata

**Affiliations:** ^1^ Division of Cardiovascular Medicine, Department of Internal Medicine Kobe University Graduate School of Medicine Kobe Japan; ^2^ Section of Arrhythmia, Division of Cardiovascular Medicine, Department of Internal Medicine Kobe University Graduate School of Medicine Kobe Japan

**Keywords:** cardiac resynchronization therapy, heart failure with reduced ejection fraction, primary prevention, ventricular arrhythmia

## Abstract

**Background:**

It is uncertain whether cardiac resynchronization therapy with a defibrillator (CRT‐D) provides better survival benefits than a CRT‐pacemaker (CRT‐P) in heart failure patients with a reduced ejection fraction (≦35%, HFrEF) treated with contemporary HF therapy.

**Methods:**

We retrospectively analyzed the ventricular arrhythmia (VAs; sustained ventricular tachycardia/fibrillation) events in HFrEF patients who underwent CRT without a prior history of VAs or aborted sudden cardiac death before the CRT implantation. Between January/2010 and December/2020, a CRT device was implanted in 79 HFrEF patients (mean age: 69 ± 12 years, male: 57, ischemic cardiomyopathy: 16). CRT‐D and CRT‐P devices were implanted in 50 and 29 patients, respectively, at each physician's discretion. CRT‐Ds were indicated in younger patients than were CRT‐Ps (66 ± 12 vs. 73 ± 12 years, *p* = 0.03), but the gender distribution did not differ (female, 24% [12 of 50] vs. 35% [10 of 29], *p* = 0.44). The VA events during a median follow‐up of 3.5‐years (interquartile range [IQR]:1.6–5.5) and their predictors were analyzed.

**Results:**

VA events occurred in 9 patients with CRT‐Ds (18%) and one with a CRT‐P (3%, *p* = 0.08). The VA event rate was significantly lower in patients without a prior non‐sustained ventricular tachycardia (NSVT: ≥3 beats; rate, ≥120 bpm; lasting <30 s, HR 0.05; 95% CI 0.01–0.30; *p* < 0.01) and females (HR 0.11; 95% CI 0.01–0.93; *p* = 0.04). Of note, no female patients without a prior history of NSVT experienced VA events.

**Conclusion:**

HFrEF CRT candidates without a prior history of NSVT and females may obtain less benefit from a primary preventive defibrillator indication.

## 1 INTRODUCTION

Cardiac resynchronization therapy (CRT) is an established treatment for patients with heart failure and a reduced ejection fraction (HFrEF).^1^ Several studies have shown the beneficial effects of CRT on both the mortality and morbidity.^2^, ^3^, ^4^, ^5^ In addition to CRT‐pacemakers (CRT‐Ps), CRT‐defibrillators (CRT‐Ds) can also deliver shocks to terminate ventricular arrhythmias (VAs; sustained ventricular tachycardia/fibrillation) and prevent sudden cardiac death (SCD). However, defibrillator shocks can be inappropriate and painful, drain the battery, increase the health care cost, and are associated with a higher risk of mortality and a reduced quality of life.^6^ On the contrary, CRT‐P devices are smaller, have a superior battery longevity, and are less expensive.^7^

Furthermore, improvements in the guideline directed medical therapy (GDMT) over the past 20 years have reduced the risk of SCD in HFrEF patients.^8^ It has also been suggested that reverse remodeling with the use of CRT reduces the risk of VAs and may contribute to a reduction in the risk of SCD.^9^ Thus, the need for an implantable cardioverter‐defibrillator (ICD) in HFrEF patients who meet the criteria for primary prevention of SCD and are eligible for CRT is often questioned. Also, there is limited data on VA events, and it is not clear whether to implant a CRTD or a CRTP in some patients. Therefore, the aim of this study was to evaluate the VA events in HFrEF patients with a CRT‐D or CRT‐P for primary prevention of SCD.

## 2 METHODS

### 2.1 Study design and patient selection

A single‐center retrospective study to analyze the VA events in HFrEF patients who underwent CRT from January 2010 to December 2020 without a prior history of sustained VAs or an aborted SCD before the CRT implantation was conducted at Kobe University Hospital in Japan. It enrolled patients eligible to receive a CRT based on the current guideline indications.^1^ Patients with an EF >35% at the time of the implant were excluded. Patients were followed for at least 1 year after the implantation. The demographics and clinical data were obtained via electronic records in all patients.

### 2.2 Echocardiography

At baseline and all the follow‐ups, the LVEF, LV end‐systolic volume (LVESV), LV end‐diastolic volume, mitral regurgitation, and interventricular dyssynchrony were assessed by echocardiography. Cardiac volumes were estimated using the modified Simpson method or Teichholz method. When only a visual assessment was available, the lower number of the LVEF range was used for the analysis.

### 2.3 Outcomes and Follow‐up

The VA events during a median follow‐up of 3.5 years (IQR:1.6–5.5) and their predictors were analyzed. VA events were defined as sustained VAs, which needed anti‐tachycardia pacing or shock therapy to terminate. Non‐sustained ventricular tachycardia (NSVT) was defined as ≧3 consecutive ventricular premature contractions at a rate of >120 bpm and lasting <30 s. A CRT volume‐responder was defined as those with an LV end‐systolic volume reduction of >15%. The data for the follow‐up assessment were obtained from the pacemaker clinic database and electronic patient records.

Outcomes of interest included the mortality and cardiac hospitalizations as tracked by the electronic patient records and databases. Cardiac hospitalizations included hospital admissions for any cardiac related event as well as unexplained syncope. Clinical follow‐up visits and device interrogations were scheduled at 1, 3, 6, and 12 months after the procedure, with remote device monitoring when possible.

### 2.4 Statistical analysis

Continuous variables are presented as either means (±standard deviation [SD]) or medians (with interquartile range [IQR]) and categorical variables as numbers and percentages (%). Differences in the continuous variables between the two groups were assessed by an unpaired t‐test or Mann–Whitney test. Categorical variables were compared through a Fisher exact test. The Kaplan–Meier method was used to estimate the survival function in patients treated with CRT. The follow‐up period from the last procedure was calculated with the median value and interquartile range. At first, to assess the clinical predictors of VT events and survival from all‐cause death, a univariate Cox proportional analysis was performed. Sequentially, variables with a *p* < 0.10 in the univariate analysis were included in the multivariate analysis, and the hazard ratios (HR) and 95% confidence interval (CI) were calculated. A value of *p* < 0.05 was considered statistically significant. All statistical analyses were performed using EZR on the R commander, version 1.36, software.

## 3 RESULTS

### 3.1 Patient characteristics

**FIGURE 1:**
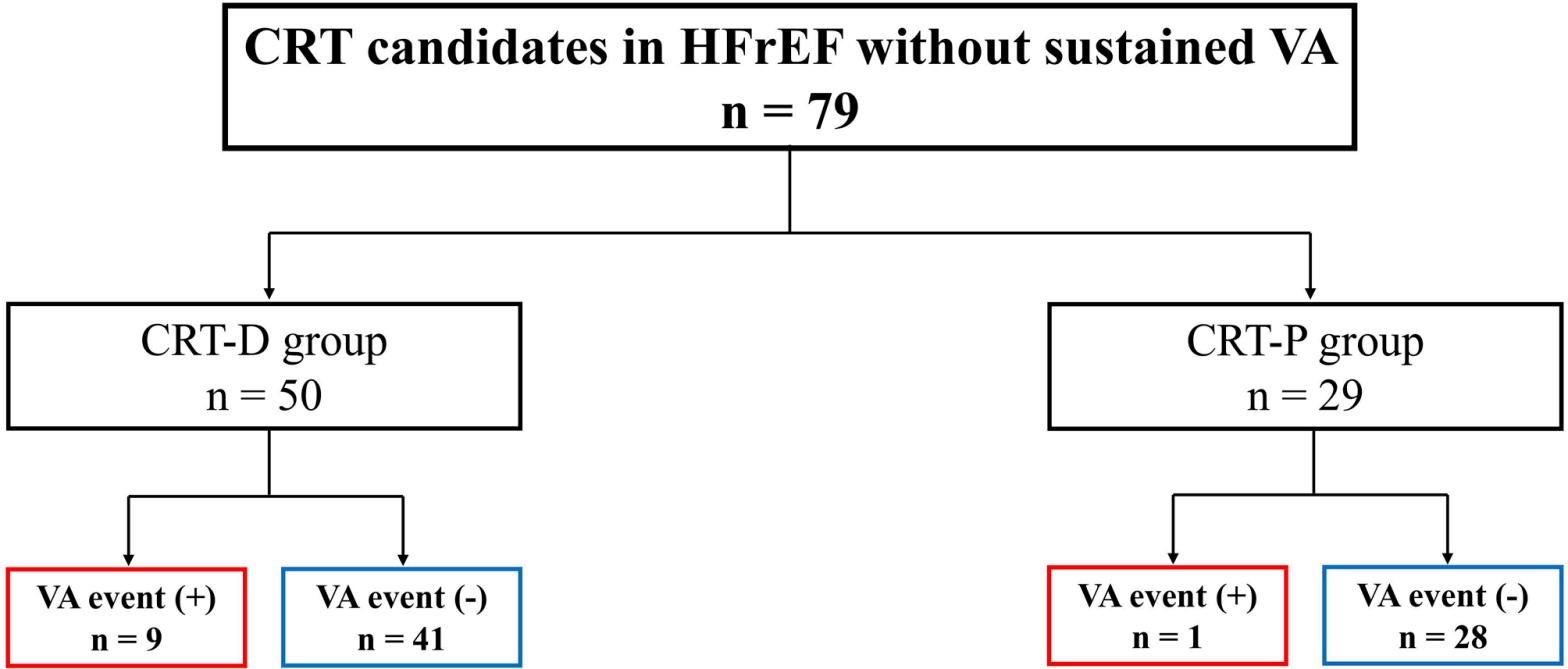
Flow chart of the protocol for the patient enrollment. CRT, cardiac resynchronization therapy; CRT‐D/‐P, cardiac resynchronization therapy with defibrillator/pacemaker; HFrEF, heart failure with reduced ejection fraction; VA, ventricular arrhythmia.

**FIGURE 2:**
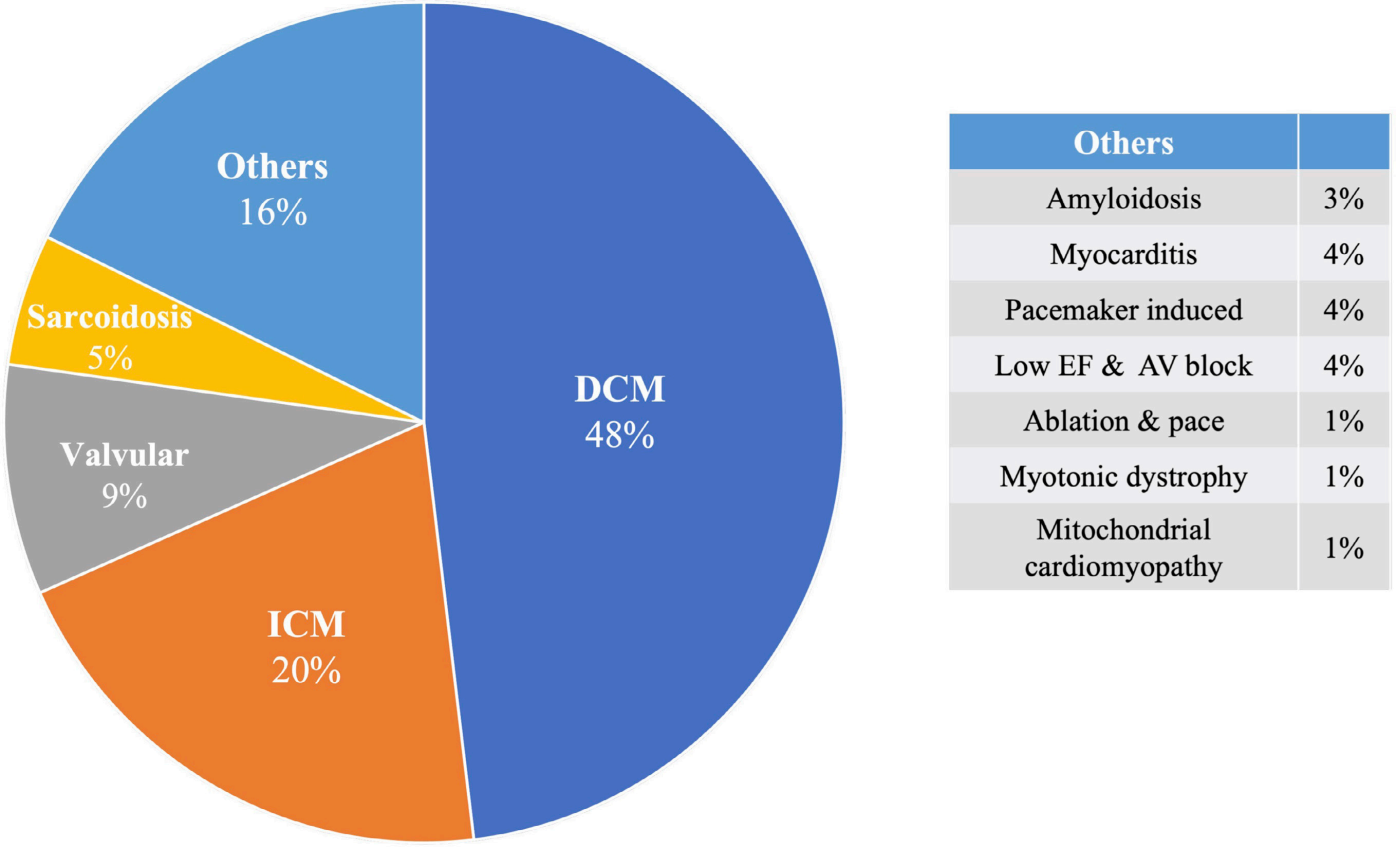
Etiology of the HFrEF patients who underwent CRT implantations. AV, atrioventricular; DCM, dilated cardiomyopathy; EF, ejection fraction; ICM, ischemic cardiomyopathy.

During the study period, 79 patients underwent CRT implantations, and CRT‐D and CRT‐P devices were implanted in 50 and 29 patients, respectively, at the physicians' discretion (Figure 1). The baseline patient characteristics are summarized in Table 1. The overall mean age was 69 ± 12 years, and 57 patients (72%) were male. The average LV ejection fraction (LVEF) was 25 ± 6%. The age and LVEF were significantly lower in the CRT‐D than CRT‐P group. The body mass index (BMI), estimated glomerular filtration rate (eGFR), and strokes were significantly lower in the CRT‐P than CRT‐D group. Patients receiving a CRT‐D were more likely to have a history of NSVT. The etiology of the HFrEF patients treated with CRT is described in Figure 2. The most frequent underlying heart disease of the HFrEF was dilated cardiomyopathy (DCM; 48%); ischemic cardiomyopathy (ICM; 20%), valvular cardiomyopathy (9%), and sarcoidosis (5%) were also common. One of 22 (4.5%) women and 15 of 57 (26.3%) men had ICM as the underlying disease.

**TABLE 1:** Patient characteristics

	CRT overall (n = 79)	CRT‐D (n = 50)	CRT‐P (n = 29)	*p* value
Demographics				
Age, (year)	69 ± 12	66 ± 12	73 ± 12	0.027
Male, n (%)	57 (72%)	38 (76%)	19 (66%)	0.435
Clinical				
LVEF, (%)	25 ± 6	24 ± 6	27 ± 6	0.034
NYHA function class III/IV, n (%)	52 (66%)	35 (70%)	17 (59%)	0.333
BNP, (pg/L)	509 ± 542	538 ± 594	459 ± 444	0.533
NSVT, n (%)	25 (32%)	21 (42%)	4 (14%)	0.012
QRS duration, (ms)	157 ± 26	155 ± 25	162 ± 25	0.208
CRT volume responder, n (%)	50 (63%)	32 (64%)	18 (62%)	1.00
eGFR, (mL/min/1.73 m^2^)	48 ± 23	53 ± 22	40 ± 23	0.017
Hemoglobin, (g/dL)	12.5 ± 1.9	12.8 ± 2.0	12.0 ± 1.4	0.049
BMI, (kg/m^2^)	22 ± 3	23 ± 3	21 ± 3	0.001
Comorbidities				
Ischemic cardiomyopathy, n (%)	16 (20%)	10 (20%)	6 (21%)	1.00
Atrial fibrillation, n (%)	38 (48%)	21 (42%)	17 (59%)	0.170
Diabetes mellitus, n (%)	27 (34%)	17 (34%)	10 (35%)	1.00
Hypertension, n (%)	32 (41%)	18 (36%)	14 (48%)	0.345
Dyslipidemia, n (%)	32 (41%)	18 (36%)	14 (48%)	0.345
Stroke, n (%)	11 (14%)	11 (22%)	0 (0%)	0.006
Smoking, n (%)	43 (54%)	30 (60%)	13 (45%)	0.243
Treatments				
Beta‐blocker, n (%)	72 (91%)	45 (90%)	27 (93%)	1.00
RASI, n (%)	63 (80%)	40 (80%)	23 (79%)	1.00
MRA, n (%)	51 (65%)	34 (68%)	17 (59%)	0.468
Prior amiodarone, n (%)	18 (23%)	13 (26%)	5 (17%)	0.419
Diuretics, n (%)	67 (85%)	43 (86%)	24 (83%)	0.751

Abbreviations: BMI, body mass index; BNP, B‐type natriuretic peptide; CRT‐D/‐P, cardiac resynchronization therapy with defibrillator/pacemaker; eGFR, estimated glomerular filtration rate (calculated by Chronic Kidney Disease Epidemiology Collaboration formula); IQR, inter‐quartile range; LVEF, left ventricular ejection fraction; MRA, mineralocorticoid receptor antagonist; NSVT, non‐sustained ventricular tachycardia; NYHA, New York Heart Association; RASI, renin–angiotensin‐system inhibitor; SD, standard deviation.

### 3.2 Clinical outcome

#### 3.2.1 VA events

During a median follow‐up period of 3.5 years (IQR, 1.6–5.5), 10 (13%) of 79 patients experienced VA events. However, the VA events did not significantly differ between the patients with an EF < 30% and those with an EF = 30–35% (HR = 0.51 [95%CI:0.14–1.82]) or CRT volume responders and non‐responders (HR = 0.41 [95%CI:0.12–1.43]) (Table 2). On the other hand, only two patients experienced inappropriate shocks due to atrial fibrillation or sinus tachycardia.

**TABLE 2:** Univariate and multivariate regression models for the prediction of VA events

	Univariate analysis		Multivariate analysis	
	HR (95% CI)	P value	HR (95% CI)	*p* value
Age (years)	1.05 (0.98 to 1.12)	0.21		
Female gender	0.11 (0.02 to 1.15)	0.06	0.11 (0.01 to 0.93)	0.04
Ischemic cardiomyopathy	1.02 (0.22 to 4.83)	0.98		
Without NSVT	0.06 (0.01 to 0.31)	<0.01	0.05 (0.01 to 0.30)	<0.01
LVEF ≦ 30%	0.51 (0.14 to 1.82)	0.30		
CRT volume responder	0.41 (0.12 to 1.43)	0.16		
Prior amiodarone	1.62 (0.42 to 6.31)	0.49		
NYHA function class III/IV	0.85 (0.23 to 3.05)	0.81		
Atrial fibrillation	1.72 (0.49 to 6.11)	0.40		
eGFR	1.00 (0.97 to 1.03)	0.81		

Abbreviations: CI, confidence interval; CRT, cardiac resynchronization Therapy; eGFR, estimated glomerular filtration rate (calculated by Chronic Kidney.Disease Epidemiology Collaboration formula); HR, hazard ratio; LVEF, left ventricular ejection fraction; NSVT, non‐sustained ventricular tachycardia; NYHA, New York Heart Association.

#### 3.2.2 Predictive factors of VA events

Table 2 shows the univariate and multivariate Cox regression analyses of the VA events. In the univariate analysis, no prior history of NSVT (HR 0.06; 95% CI 0.01–0.31; *p* < 0.01) was associated with fewer VA events. On the other hand, ICM (HR 1.02; 95% CI 0.22–4.83; *p* = 0.98) was not an independent predictor of VA events. A Cox proportional model was used for the multivariate analysis, and variates with a *p* < 0.10 in the univariate analysis were included in the model. A female gender (HR 0.11; 95% CI 0.01–0.93; *p* = 0.04) and no prior history of NSVT (HR 0.05; 95% CI 0.01–0.30; *p* < 0.01) were independent predictors of fewer VA events in the multivariate analysis. Furthermore, all female patients without a prior history of NSVT experienced no VA events.

#### 3.2.3 Mortality

**FIGURE 3:**
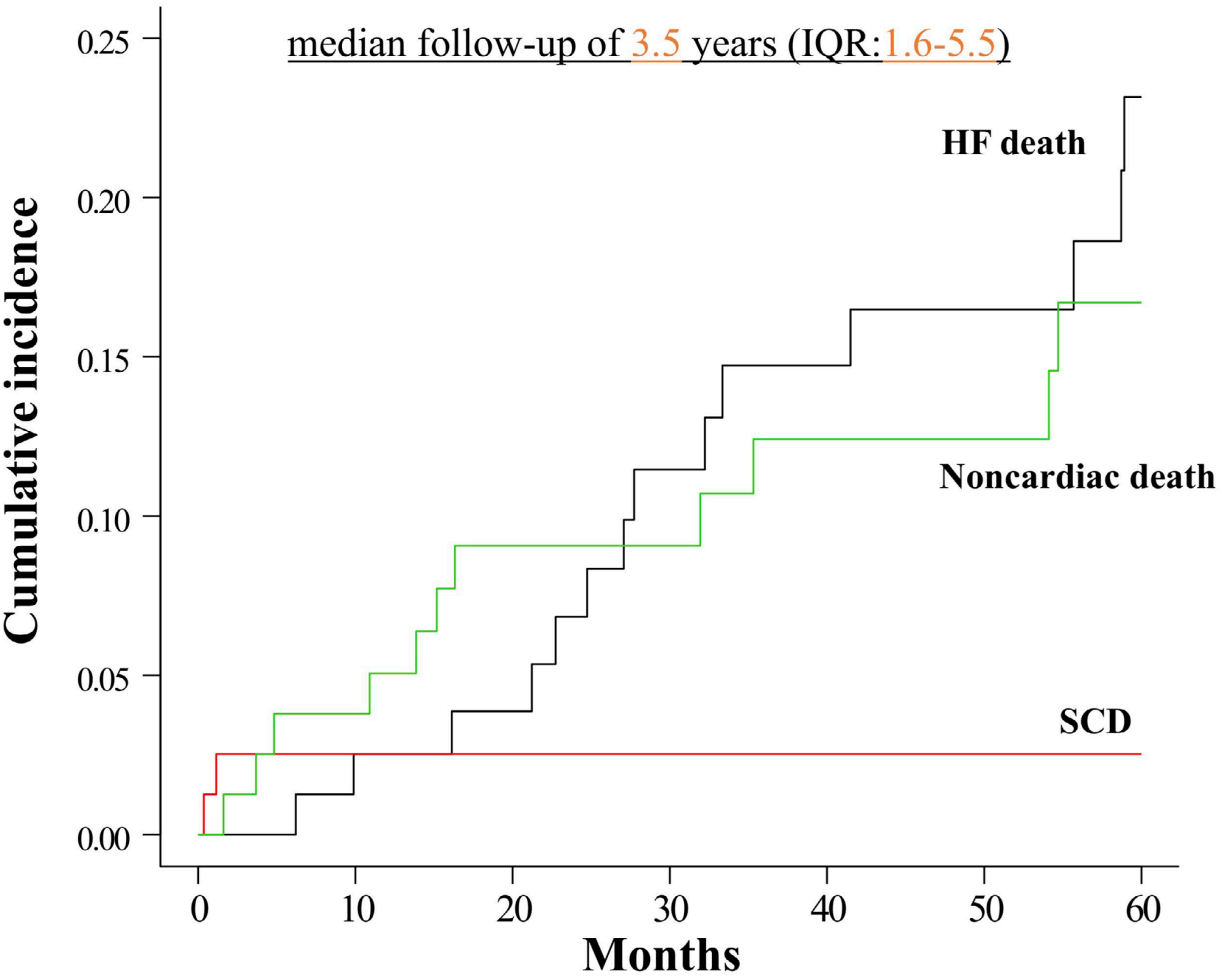
Cumulative incidences of the different types of death. IQR, interquartile range; HF, heart failure; SCD, sudden cardiac death.

Thirty‐two patients (41%) died during the follow‐up. During a median follow‐up period of 3.5 years, the cumulative incidence of deaths due to HF was 23.1% (95% CI 13.2 to 34.7); the incidences of noncardiac death and SCD were 16.7% (95% CI 8.7 to 27.0) and 2.5% (95% CI 0.5 to 7.9), respectively (Figure 3). The cause of death was SCD in 2 patients (6%; 1 with a CRT‐D and 1 with a CRT‐P), terminal HF in 14 (44%; 11 with a CRT‐D and 3 with a CRT‐P), and noncardiac death in 16 (50%; 10 with a CRT‐D and 6 in with a CRT‐P). Three (all with a CRT‐D) out of 14 patients with an HF death and 2 (all in CRT‐D) out of 16 with a noncardiac death experienced VA events, respectively. Table 3 shows the Cox regression analyses of the all‐cause mortality. Age was associated with an increased risk of all‐cause mortality (HR 1.05; 95% CI 1.00–1.11; *p* = 0.049), and no prior history of NSVT (HR 0.40; 95% CI 0.18–0.86; *p* = 0.018) and the eGFR (HR 0.96; 95% CI 0.94–0.99; *p* < 0.01) were associated with a decreased risk of all‐cause mortality in the multivariate analysis.

**TABLE 3:** Univariate and multivariate regression models for the prediction of all‐cause death

	Univariate analysis		Multivariate analysis	
	HR (95% CI)	*p* value	HR (95% CI)	*p* value
Age (years)	1.07 (1.02 to 1.12)	<0.01	1.05 (1.00 to 1.11)	0.049
Female gender	0.52 (0.22 to 1.26)	0.15		
Ischemic cardiomyopathy	1.95 (0.92 to 4.12)	0.08	0.64 (0.26 to 1.57)	0.33
Without NSVT	0.35 (0.17 to 0.74)	<0.01	0.40 (0.18 to 0.86)	0.018
LVEF ≦ 30%	1.21 (0.52 to 2.80)	0.66		
CRT volume responder	0.37 (0.18 to 0.75)	<0.01	0.54 (0.25 to 1.16)	0.11
Prior amiodarone	1.32 (0.59 to 3.00)	0.50		
NYHA function class III/IV	2.43 (1.00 to 5.92)	0.05	1.64 (0.63 to 4.24)	0.31
Atrial fibrillation	1.98 (0.97 to 4.056)	0.06	0.93 (0.43 to 2.04)	0.86
eGFR	0.96 (0.94 to 0.98)	<0.01	0.96 (0.94 to 0.99)	<0.01

Abbreviations: CI, confidence interval; CRT, cardiac resynchronization Therapy; eGFR, estimated glomerular filtration rate (calculated by Chronic Kidney.Disease Epidemiology Collaboration formula); HR, hazard ratio; LVEF, left ventricular ejection fraction; NSVT, non‐sustained ventricular tachycardia; NYHA, New York Heart Association.

## 4 DISCUSSION

### 4.1 Main findings

We evaluated the VA events in HFrEF patients who were implanted with a CRT‐D or CRT‐P for primary prevention. The main findings of our study were:

1. During a median follow‐up period of 3.5 years (IQR, 1.6–5.5), 13% of the patients who were implanted with a CRT‐D/CRT‐P for primary prevention experienced VA events.
2. A “female gender” and “no prior history of NSVT” were independent predictors of fewer VA events in the multivariate analysis.
3. There was no difference in the VA event and mortality rates between the CRT‐D and CRT‐P groups.

### 4.2 Gender differences in VA events and mortality

In the present study, VA events occurred significantly less often in women than men. However, there was no difference in the all‐cause death rates between men and women. There was a potentially lesser benefit of primary prevention ICDs in women, because SCD may have a smaller impact on the total mortality in women than in men. A previous study showed that women receive significantly fewer appropriate ICD shocks.^10^, ^11^ Nevertheless, you must pay attention to the sex differences in the baseline characteristics and comorbidities. In fact, the distribution of ICM in the present study was not equal between the women and men (4.5% vs. 26%).

How sex differences translate into clinical arrhythmias and possibly appropriate ICD shocks has not been fully studied. There were sex differences in the electrophysiological properties such as repolarization, calcium handling, autonomic modulation, and ion channels.^12^ It also cannot be ruled out whether there is a bias in that physicians tend to withhold ICD‐therapy from women with a worse prognosis. An additional factor may be that women, in general, have a longer life expectancy than men.

### 4.3 The impact of NSVT on VA events

Identifying patients at high risk of SCD remains a challenge. Many studies have investigated the predictive value of NSVT for SCD, but the results are ambiguous. Some studies have shown that NSVT does not increase the risk of SCD and all‐cause mortality^13^; however, other studies have demonstrated that NSVT is an independent risk factor of SCD or sustained VAs.^14^, ^15^ In the present study, no history of NSVT was a less predictive independent risk factor for VA events after a CRT implantation. Nevertheless, the frequency of NSVT and the tachycardia cycle length were not studied. Generally, NSVT and sustained VAs are based on electrical instability, but sustained VAs represent a more severe electrophysiological condition. With the continued deterioration of the condition, the number and duration of NSVT episodes may increase and even progress to sustained VAs. Thus, there may be a temporal relationship between NSVT and sustained VAs.

### 4.4 Clinical implications

The question of whether patients with HFrEF should receive a CRT‐D or CRT‐P is one of the major dilemmas in routine clinical practice in cardiovascular medicine. Especially given the physique and BMI of older Asian women, doctors are hesitant to implant physically large devices, like a CRT‐D or ICD. In this real‐world data, only 1 patient with a CRT‐P experienced SCD. The result of the present study demonstrated that our choice of CRT devices would have been acceptable. Furthermore, “no previous history of NSVT” and a “female gender” may be factors that are less likely to cause events after a CRT implantation. Nevertheless, the latest Japanese data from a sub‐analysis of the Nippon Storm study, demonstrated that the rate of appropriate ICD therapy is commonly applied at comparable rates for either primary or secondary prophylaxis in ICM patients with HFrEF.^16^ Thus, it is believed that adapting the results of our study to patients with ICM may have a high‐risk. Also, some recent large observational studies demonstrated that CRT‐D is associated with a significant risk reduction in all‐cause mortality as compared to CRT‐P in patients with ICM, but not in those with non‐ICM.^17^, ^18^ In fact, according to the latest European guidelines, primary ICD indications are being reviewed for non‐ICM.^19^ A direct randomized comparison of CRT‐Ds with CRT‐Ps in patients with HFrEF remains the only way to answer this major unsolved question in cardiovascular medicine. Fortunately, such attempts are underway. A major project in this field is the RESET‐CRT clinical trial (ClinicalTrials.gov number NCT03494933) that will randomize HFrEF and a CRT indication to either a CRT‐D or CRT‐P implantation. The randomized RESET‐CRT trial will provide urgently needed evidence in this debated field.

In clinical practice, the GDMT for HFrEF has improved dramatically. Patients without contraindications appear to gain the most benefit from a combined treatment with the ‘fantastic four’: an angiotensin receptor/neprilysin inhibitor (ARNI), beta‐blocker, MRA, and sodium–glucose co‐transporter 2 (SGLT2) inhibitor.^20^ We believe that it would be necessary to evaluate the prognosis after the CRT implantation in patients who comply with the current GDMT.

### 4.5 Study limitations

First, this study was a single‐center retrospective observational analysis and the number of study patients was relatively small. However, the observation period was considered to be adequate to analyze the ventricular arrhythmia events after CRT implantations (median 3.5 years, IQR 1.6–5.5 years). Additional multicenter, randomized trials should be performed to obtain a more objective assessment. Second, this study included patients with ICM and non‐ICM, and the non‐ICM consisted of a non‐uniform etiology. Moreover, sex differences in the distribution of ICM are also a limitation of the present study. Nevertheless, all the consecutive patients who underwent CRT implantations at our hospital met the inclusion criteria (LVEF<35 and primary prevention of SCD), which would have reduced the potential selection bias. Third, there was a possibility that the differences in the baseline characteristics between the CRT‐D and CRT‐P groups affected the incidence of VA events and the mortality rate after the CRT implantation. Fourth, whether to implant a CRT‐D or CRT‐P was left to the physicians' discretion in the present study. Further prospective studies are needed to elucidate this issue. Fifth, the detection and therapy programming in the tachycardia zones depended on each patient. Sixth, there was no uniformity in how and when the history of NSVT in each patient was determined. As the previous study showed, frequent monitoring would be more reliable to identify the importance of NSVT before a device implantation.^21^ Finally, most patients were not prescribed novel heart failure drugs, such as an ARNI or SGLT2 inhibitors, further studies are needed to evaluate the effect of these medications on the ventricular arrhythmia events in CRT patients. However, this study was conducted in a group of patients who were equally and appropriately treated according to the guidelines of their time. Therefore, the results of this study would provide one clue to estimate the factors that contribute to the occurrence of ventricular arrhythmias after CRT implantations in a group of patients who are appropriately receiving the latest or future updated heart failure treatment guidelines.

## 5 CONCLUSIONS

In a contemporary real‐world cohort of patients with HFrEF treated with CRT and with an indication for a primary prevention ICD, the HFrEF CRT candidates without a prior history of NSVT or that were female may have gained less benefit from a primary prevention defibrillator indication. These data may support the decision‐making as to whether to implant a CRT‐D or CRT‐P in HFrEF patients for primary prevention of SCD.

## DECLARATIONS

*Approval of the research protocol*: The study was performed in compliance with the principles outlined in the Declaration of Helsinki and approved by the ethics committee of Kobe University Hospital (Committee of 25 April 2022, Approval No. 220009).

### Informed consent

The patients consented to the use of their anonymized clinical data for research purposes by the opt‐out method.

### Registry and the registration No

N/A.

### Animal studies

N/A.

## AUTHOR CONTRIBUTIONS

Toshihiro Nakamura: clinical practice/data sampling/drafting article. Koji Fukuzawa: concept/design/data analysis/interpretation/drafting article. Kunihiko Kiuchi: data collection/statics. Mitsuru Takami: data collection/statics. Yusuke Sonoda: data collection/statics. Hiroyuki Takahara: data collection/statics. Kazutaka Nakasone: data collection/statics. Kyoko Yamamoto: data collection/statics. Yuya Suzuki: data collection/statics. Ken‐ichi Tani: data collection/statics. Hidehiro Iwai: data collection/statics. Yusuke Nakanishi: data collection/statics. Mitsuhiko Shoda: data collection/statics. Atsushi Murakami: data collection/statics. Shogo Yonehara: data collection/statics. Ken‐ichi Hirata: Supervision.

## FUNDING INFORMATION

This research did not receive any specific grant from funding agencies in the public, commercial, or not‐for‐profit sectors.

## CONFLICT OF INTEREST

The Section of Arrhythmia is supported by an endowment from Abbott JAPAN, Medtronic JAPAN, and Boston Scientific JAPAN. Ken‐ichi Hirata chairs the Section, and Koji Fukuzawa and Mitsuru Takami belong to the Section. However, the authors have no competing interests to declare that are relevant to the content of this article.
